# A method for concentrating lipid peptide DNA and siRNA nanocomplexes that retains their structure and transfection efficiency

**DOI:** 10.2147/IJN.S78935

**Published:** 2015-04-01

**Authors:** Aristides D Tagalakis, Sara Castellaro, Haiyan Zhou, Alison Bienemann, Mustafa M Munye, David McCarthy, Edward A White, Stephen L Hart

**Affiliations:** 1 Experimental and Personalised Medicine Section, University College London (UCL) Institute of Child Health, London, UK; 2 Department of Pharmacy, University of Genova, Genova, Italy; 3 Functional Neurosurgery Research Group, School of Clinical Sciences, AMBI Labs, University of Bristol, Southmead Hospital, Bristol, UK; 4 UCL School of Pharmacy, London, UK

**Keywords:** nanoparticles, concentration, anionic liposome, siRNA, DNA, targeted gene delivery

## Abstract

Nonviral gene and small interfering RNA (siRNA) delivery formulations are extensively used for biological and therapeutic research in cell culture experiments, but less so in in vivo and clinical research. Difficulties with formulating the nanoparticles for uniformity and stability at concentrations required for in vivo and clinical use are limiting their progression in these areas. Here, we report a simple but effective method of formulating monodisperse nanocomplexes from a ternary formulation of lipids, targeting peptides, and nucleic acids at a low starting concentration of 0.2 mg/mL of DNA, and we then increase their concentration up to 4.5 mg/mL by reverse dialysis against a concentrated polymer solution at room temperature. The nanocomplexes did not aggregate and they had maintained their biophysical properties, but, importantly, they also mediated DNA transfection and siRNA silencing in cultured cells. Moreover, concentrated anionic nanocomplexes administered by convection-enhanced delivery in the striatum showed efficient silencing of the β-secretase gene *BACE1*. This method of preparing nanocomplexes could probably be used to concentrate other nonviral formulations and may enable more widespread use of nanoparticles in vivo.

## Introduction

Nonviral formulations comprising mixtures of cationic polymers or lipids that self-assemble into nanoparticles upon mixing with nucleic acids have been widely used in genetic and gene therapy research since the late 1980s. More recently, these vectors have become more prevalent with the development of small interfering RNA (siRNA)-mediated gene silencing.^1^–^8^ While a wide variety of formulations are used in cell culture experiments, the number of formulations in clinical trials for gene or siRNA therapies is more limited.^8^ The barriers to transfection in vivo^8^,^9^ are very different to the cell culture environment and not all formulations are compatible with usage in live animals. A further challenge is the technical problems associated with preparing monodisperse nanoparticle formulations in the volumes and concentrations required in vivo.^10^

Self-assembly of nanoparticles with cationic reagents and nucleic acids is mediated largely by electrostatic interactions. Empirically optimized ratios of these components are required to produce monodisperse nanoparticles with peak transfection efficiency. The formulation used in this study comprises a mixture of liposomes and targeting peptides that self-assemble on mixing with DNA^11^–^18^ or siRNA^19^–^21^ to form LPD or LPR receptor-targeted nanocomplexes (RTNs). Based on this platform, we have described both cationic and anionic RTN formulations and shown their in vivo potential by delivery to tumors, lung epithelium, brain, and blood vessels.^11^–^16^,^18^,^21^,^22^ The formulation of RTNs is more complicated than simple lipoplex or polyplexes and becomes increasingly problematic in our experience at higher nucleic acid concentrations of more than 0.5 mg/mL with large aggregates and precipitates forming, which leads to poor biodistribution, systemic toxicity, and low transfection efficiency in vivo.^10^

The RTN formulation process requires fast mixing of liposome, peptide, and nucleic acid components, which is easily achieved by rapid pipetting at low volumes and concentrations – for example, where the nucleic acid is less than 300 μg/mL. For in vivo applications by systemic delivery, or in the brain where only very small volumes can be injected, higher concentrations of nucleic acids are required of 1 mg/mL or more.^10^,^23^ At these concentrations, nanocomplexes following pipette-based mixing methods, even in small volumes, rapidly precipitate due to instantaneous electrostatic interactions of the components. Sophisticated mixing technologies are available such as microfluidics, continuous flow systems, and in-line mixing,^24^–^27^ but each of these usually requires expensive equipment and time-consuming optimization of the process and consumes large amounts of materials increasing costs.

Recently, Vauthier et al^28^ proposed a method of concentrating nanoparticles without aggregation using osmosis in a simple laboratory setup. We report here the application of this facile, small-scale method to concentrate LPD and LPR formulations, which should work equally well with liposomal or polymeric nanocomplex formulations, enhancing their utility for in vivo experiments. Nanocomplexes were evaluated for their biophysical properties, as well as for their in vitro and in vivo transfection efficiencies. This method could find widespread utility in the development of many different nucleic acid formulations for in vivo applications, potentially hastening the development of new genetic therapies.

## Materials and methods

### Materials

Dextran (molecular weight [MW] 150,000) from *Leuconostoc mesenteroides* and dialysis tubing cellulose membrane MW cut-off (CO) 14,000 were obtained from Sigma-Aldrich Co. (St Louis, MO, USA). 1,2-dioleoyl-sn-glycero-3-phospho-(1′-rac-glycerol) (DOPG), 1,2-di-O-octadecenyl-3-trimethylammonium propane (DOTMA), 1,2-dioleoyl-sn-glycero-3-phosphoethanolamine (DOPE), 1,2-dipalmitoyl-sn-glycero-3-phosphoethanolamine-N-(methoxy[polyethylene glycol]-2000) (DPPE PEG2000), and DOTMA/DOPE (1:1 molar ratio) were purchased from Avanti Polar Lipids, Inc. (Alabaster, AL, USA). Peptide Y (K_16_GACYGLPHKFCG) was synthesized by ChinaPeptides Co., Ltd. (Shanghai, People’s Republic of China). Rabies virus glycoprotein targeting peptide (RVG-9R) (YTIWMPENPRPGTPCDIFTNSRGKRASNGGGG-RRRRRRRRR) was synthesized by AMS Biotechnology Limited (Abingdon, UK). Silencer Firefly Luciferase (GL2 + GL3) and Silencer Negative Control #1 siRNA were obtained from Applied Biosystems (Thermo Fisher Scientific, Waltham, MA, USA). Lipofectamine^®^ 2000 (L2K) was purchased from Invitrogen (Thermo Fisher Scientific). The siRNA for the *BACE1* in vivo studies was bought from Eurofins MWG Operon LLC (Huntsville, AL, USA) and the sequences were: *BACE1* (sense) 5′ GCUUUGUGGAGAUGGUGGAdTdT 3′; and *BACE1* (antisense) 5′ UCCACCAUCUCCACAAAGCdTdT 3′. The plasmid pCI-Luc consists of the luciferase gene from pGL3 (Thermo Fisher Scientific) subcloned into pCI (Promega Corporation, Fitchburg, WI, USA). The plasmid pEGFP-N1 (4.7 kb) containing the gene *GFP* was obtained from Clontech Laboratories, Inc. (Mountain View, CA, USA).

### Liposome and nanoparticle formulation

Lipid stocks were dissolved in chloroform at 10 mg/mL. Lipids were then mixed at the required molar ratios in a round-bottomed flask and the chloroform was slowly evaporated in a rotary evaporator (BÜCHI Labortechnik AG, Flawil, Switzerland) to produce a lipid film. Lipids were then rehydrated with sterile, distilled water while constantly rotated overnight, and they were then sonicated in an ultrasonic water bath (Jencons-PLS, Bedfordshire, UK) to reduce their size. The anionic liposomes that were made were: DOPG:DOPE:DOPE PEG2000 (L^AP^1); and DOPG:DOPE:DPPE PEG2000 (L^AP^2) at a molar ratio of 47.5:47.5:5 mol%, respectively.^16^,^21^

Anionic nanocomplexes were prepared in water at a 4:3:1 molar charge ratio^16^ of liposome:peptide:siRNA or liposome:peptide:DNA, by adding the peptide to the siRNA (PRL) or DNA (PDL), incubating for 15 minutes at room temperature, and then adding the liposome with rapid mixing and incubating at room temperature for a further 20 minutes. Cationic RTN formulations (at a weight ratio of 1:4:1, liposome:peptide:DNA) were made by first adding the peptide to the liposome DOTMA/DOPE, followed by addition of the DNA with rapid mixing and incubation for 30 minutes at room temperature to allow for complex formation. The nanocomplexes prepared were termed LYD (liposome DOTMA/DOPE, peptide Y, and DNA), PRL (peptide Y or RVG-9R, siRNA, liposome L^AP^2), and PDL (peptide Y, DNA, liposome L^AP^1). Peptide/siRNA nanocomplexes were also made by mixing peptide RVG-9R with siRNA at a 4:1 weight ratio and incubation at room temperature for 30 minutes.

### Concentration experiments by reverse dialysis

The dialysis tubing was boiled in a solution of 2% NaHCO_3_/1 mM ethylenediaminetetraacetic acid (EDTA) for 10 minutes. It was then rinsed thoroughly with ddH_2_O and then boiled for 10 minutes in a solution of 1 mM EDTA. It was then rinsed with ddH_2_O, cooled at room temperature, and then stored at 4°C in a fresh solution of 1 mM EDTA. Dextran solutions (100–300 g/L) were prepared by dissolving small amounts of dextran in sterile ddH20 by stirring and heating at 60°C for 30 minutes.

Then, 0.5–1.5 mL of nanocomplexes were prepared as described earlier at a nucleic acid concentration of 175–200 μg/mL. The volume of the counter-dialysis (dextran) medium was 12–14 mL (depending on the volume of the nanocomplexes in the tubing), and different concentrations of dextran were used: 100 g/L; 150 g/L; 200 g/L; 250 g/L; and 300 g/L. Dialysis was performed at room temperature in disposable conical centrifuge 15 mL tubes. At different time points, and at the end of the dialysis, the concentrated nanocomplexes were collected from the dialysis tubing and the concentration was measured in a NanoDrop spectrophotometer (Thermo Fisher Scientific), and the size and charge measurements were performed, as will be described. Each experiment was performed in triplicate.

### Particle size and charge measurements

Nanocomplex preparations were diluted with distilled water to a final volume of 1 mL at a concentration of 5 μg/mL with respect to DNA or siRNA. They were then analyzed for size by intensity and charge (ζ potential) by dynamic light scattering (DLS) using a Malvern Nano ZS Zetasizer (Malvern Instruments, Malvern, UK). The data were then processed by software provided by the manufacturer, DTS version 5.03. Size measurements with a polydispersity index (PDI) of less than 0.3 were accepted as monodisperse.

### In vitro transfections

The murine neuroblastoma cell line Neuro-2A (American Type Culture Collection, Manassas, VA, USA) and Neuro-2A-Luc cells, were maintained in Dulbecco’s Modified Eagle’s Medium, 1% nonessential amino acids, 1 mM sodium pyruvate, and 10% fetal calf serum (FCS) (Thermo Fisher Scientific). The human bronchial epithelial cells 16HBE14o–(shortened to HBE) were provided by D Gruenert (San Francisco, CA, USA) and were cultured in Eagle’s Minimal Essential Medium with HEPES modification (Sigma-Aldrich Co.), 10% FCS, and 2 mM L-glutamine. All cells were maintained at 37°C in a humidified atmosphere in 5% carbon dioxide. Cells were seeded in 96-well plates at 2×10^4^ cells per well 24 hours prior to transfection. Following the removal of growth medium, 200 μL of complexes that underwent concentration were diluted in OptiMEM in order to contain 0.25 μg of plasmid DNA or 50 nM siRNA, and they were added to the cells in replicates of six. The same amount of DNA or siRNA was added per well when the nanocomplexes were made fresh (ie, not concentrated). Plates were centrifuged at 1,500 rpm for 5 minutes (400× *g*) and incubated for 4 hours at 37°C, then transfection medium was replaced by the complete growth medium and incubated for a further 24 hours. Luciferase expression was measured in cell lysates with a luciferase assay (Promega Corporation) in a FLUOstar OPTIMA luminometer (BMG LABTECH GmbH, Ortenberg, Germany). The amount of protein present in each sample was determined with the Bio-Rad protein assay reagent (Bio-Rad Laboratories Inc., Hercules, CA, USA) in a FLUOstar OPTIMA luminometer. Luciferase activity was expressed as relative light units per milligram of protein (RLU/mg). Each measurement was performed in groups of six and the mean was determined.

Neuro-2A cells were seeded in 96-well plates at 1.2×10^4^ cells per well 24 hours prior to transfection with 175 μL of complete serum-containing media. Twenty-four hours later, 25 μL of concentrated PDL complexes were diluted in OptiMEM to contain 0.25 μg of green fluorescent protein (GFP) plasmid DNA and then added to the cells in replicates of six. Plates were centrifuged at 1,500 rpm for 5 minutes (400× *g*) and incubated for 48 hours at 37°C. They were then imaged (20× magnification) using an Olympus IX70 fluorescent microscope (Olympus Corporation, Tokyo, Japan).

### Transmission electron microscopy (TEM)

Copper grids (300-mesh) coated with a Formvar/carbon support film (Agar Scientific Ltd, Stansted, UK) were prepared by glow discharge in an Emitech K350G system (Emitech LTD, Nicosia, Cyprus). Nanocomplex preparations were applied to grids as a droplet then after 5 seconds, the grid was dried by blotting with filter paper. The sample was then negatively stained with 1% uranyl acetate for 2–3 seconds, before blotting with filter paper and air-dried. Imaging was performed with a Philips CM120 BioTwin TEM and operated at an accelerating voltage of 120 kV. The images were captured using an AMT 5 MP digital TEM camera (Deben UK Limited, Suffolk, UK).

### Cell proliferation assay

Cell viability was assessed using the CellTiter 96^®^ Aqueous One Solution Cell Proliferation Assay (Promega Corporation). Neuro-2A cells were seeded in 96-well plates and transfected with anionic and cationic concentrated nanocomplexes that were diluted in OptiMEM, as discussed earlier. After 24 hours, the media were changed with growth media containing 20 μL of CellTiter 96 Aqueous One Solution reagent. Finally, after incubation for 2 hours, absorbance was measured at 490 nm on a FLUOstar OPTIMA spectrophotometer (BMG LABTECH GmbH). Cell viability for each complex was expressed as a percentage of the viability of control cells.

### Ex vivo silencing study using RVG-9R peptide

Brains were obtained from wild-type CD1 mice and cut into small pieces (~2 mm ×2 mm ×2 mm) in a Petri dish under sterile conditions, then moved to a 24-well plate by forceps. Then, 1 mL of DMEM (1% antibiotics, no serum) was added to each well and incubated at 37°C and transfected in triplicate, as described previously. Forty-eight hours later, the medium was removed from the culture dish and the tissues were processed for RNA extraction using the RNeasy kit according to the manufacturer’s instructions (Qiagen NV, Venlo, the Netherlands).

### In vivo procedures

All murine in vivo studies were performed in accordance with University College London’s animal care policies and with the authority of the appropriate UK Home Office licenses. CD1 female mice (6–8 weeks old) were injected intravenously with 100 μL of anionic PRL nanoparticles (with peptide RVG-9R) containing 16 μg or 50 μg of siRNA (0.64–2 mg/kg) in 5% dextrose. Mice were euthanized 48 hours later and their brains were placed in RN Alater (Thermo Fisher Scientific). All rat in vivo studies were performed in accordance with the University of Bristol’s animal care policies and with the authority of the appropriate UK Home Office licenses. Adult male Wistar rats (Charles River Laboratories International, Inc., Wilmington, MA, USA) (225–275 g) were used for convection-enhanced delivery (CED), as described previously.^15^,^21^ A total volume of 5 μL (6 μg of siRNA or 0.024 mg/kg) of anionic PRL nanoparticles (with peptide RVG-9R) in 5% dextrose was delivered to the striatum at an infusion rate of 2.5 μL/minute. Rats were euthanized 48 hours later, and the striata were placed in RNAlater (Thermo Fisher Scientific).

### qRT-PCR

Total RNA was extracted from rat brain using the RNeasy kit according to the manufacturer’s instructions (Qiagen NV). RNA was checked for integrity using the Agilent 2100 Bioanalyzer (Agilent Technologies, Santa Clara, CA, USA). All RNA samples had an RNA integrity number of more than 8, indicating high-quality RNA. Prior to reverse transcription, each RNA sample was treated with DNase (Thermo Fisher Scientific). First-strand complementary DNA was synthesized from 1 μg of DNase-treated RNA, using random hexamers, and Superscript II reverse transcriptase (Thermo Fisher Scientific) in a 1-hour reaction at 37°C. Rat BACE1 and rat beta-actin complementary DNAs were then quantified using Taqman primers and probes (Rn00569988_m1 and Rn00667869_m1, respectively; Thermo Fisher Scientific) and an ABI PRISM^®^ 7000 Sequence Detection System (Applied Biosystems; Thermo Fisher Scientific). The quantitative reverse transcription polymerase chain reaction (qRT-PCR) assay conditions were: stage 1, 50°C for 2 minutes; stage 2, 95°C for 10 minutes; stage 3, 95°C for 15 seconds, then 60°C for 1 minute; repeated 40 times.

### Western blot

Protein gel electrophoresis and Western blot analysis were performed as described previously.^21^ Briefly, total protein was extracted from mouse brain using the Precellys^®^ Homogenizer (Stretton Scientific Ltd, Derbyshire, UK). Forty micrograms of protein were loaded and separated using NuPAGE^®^ Precast gels (10% Bis-Tris; Thermo Fisher Scientific) and then transferred electrophoretically to a polyvinylidene fluoride membrane (Thermo Fisher Scientific). The primary antibodies used in this study were rabbit anti-BACE1 polyclonal antibody (EE-17, 1:1,000; Sigma-Aldrich Co.) and mouse anti-β-tubulin monoclonal antibody (1:5,000; Sigma-Aldrich Co.), and the secondary antibodies were a horseradish peroxidase-conjugated antimouse or antirabbit immunoglobulin G (Stratech Scientific Ltd, Suffolk, UK) (1:50,000). Semiquantification of the bands was performed by densitometry using the ImageJ software.

### Statistical analysis

The data presented in this study are expressed as the mean ± standard deviation and were analyzed using a two-tailed, unpaired Student’s *t*-test or one-way analysis of variance and Bonferroni’s post hoc analysis, where applicable.

## Results and discussion

### Concentrating nanoparticles and biophysical characterization

**Figure 1:**
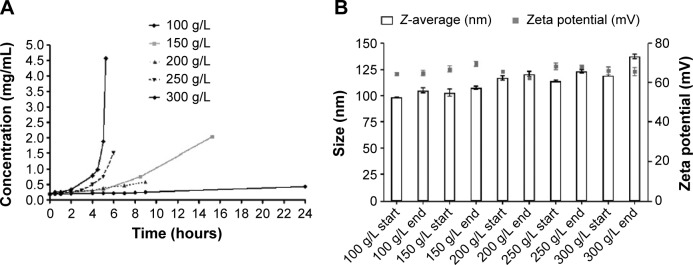
Effect of different concentrations of dextran on concentrating cationic LYD nanoparticles and on size and zeta potential. **Notes:** (**A**) Kinetics of the concentration of LYD nanoparticles over time when varying the concentration of dextran (100–300 g/L). (**B**) Size and charge measurements of LYD nanoparticles by dynamic light scattering before and after concentration with different amounts of dextran (100–300 g/L). **Abbreviation:** LYD, liposome 1,2-di-O-octadecenyl-3-trimethylammonium propane (DOTMA)/1,2-dioleoyl-sn-glycero-3-phosphoethanolamine (DOPE), peptide Y, and DNA.

Concentration of LYD nanoparticles^19^ by reverse dialysis was first performed through cellulose dialysis membrane (MWCO: 14,000) against solutions of dextran (MW: 150,000) dissolved in a range of concentrations. The high osmotic pressure of the dextran solutions leads to the displacement of water from the tubing into the surrounding dextran solution. Figure 1A shows the change in concentration of nanocomplexes achieved over time from a starting concentration of 0.2 mg/mL with respect to the DNA with varying dextran concentrations. The lowest dextran concentration of 100 g/L resulted in a 30% increase in nanocomplex concentration after 9 hours of dialysis, whereas the 150 g/L, 200 g/L, 250 g/L, and 300 g/L concentrations resulted in 3.9-fold (in 8.5 hours), 2.7-fold (in 9 hours), 8.2-fold (in 6 hours), and 23.9-fold (in 5 hours and 15 minutes) increases, respectively. When longer time points were assessed for dextran at 100 g/L (24 hours) and 150 g/L (15 hours and 15 minutes), 2.25-fold and 10.6-fold increases in DNA concentration were achieved, respectively. At dextran concentrations of >150 g/L nanoparticle concentrations of 0.59–4.6 mg/mL were achieved, which is in the target range for possible clinical applications.^10^,^29^

The effect of the starting volume at three different concentrations of dextran (100 g/L, 200 g/L, and 300 g/L) was investigated for its effects on the rate of concentration increase. Table 1 shows that smaller starting volumes were concentrated more quickly. For example, at 300 g/L, a ~9-fold concentration of DNA in a 0.5 mL formulation was achieved in only 2.2 hours, while 4.5 hours were required for the equivalent 1.5 mL formulation (Figure 1A). Therefore, the osmotic pressure applied to the nanoparticle solution could be controlled through the dextran concentration in the counter-dialyzing solution, which significantly affected the rate of water transfer between the two compartments.

**Figure 2:**
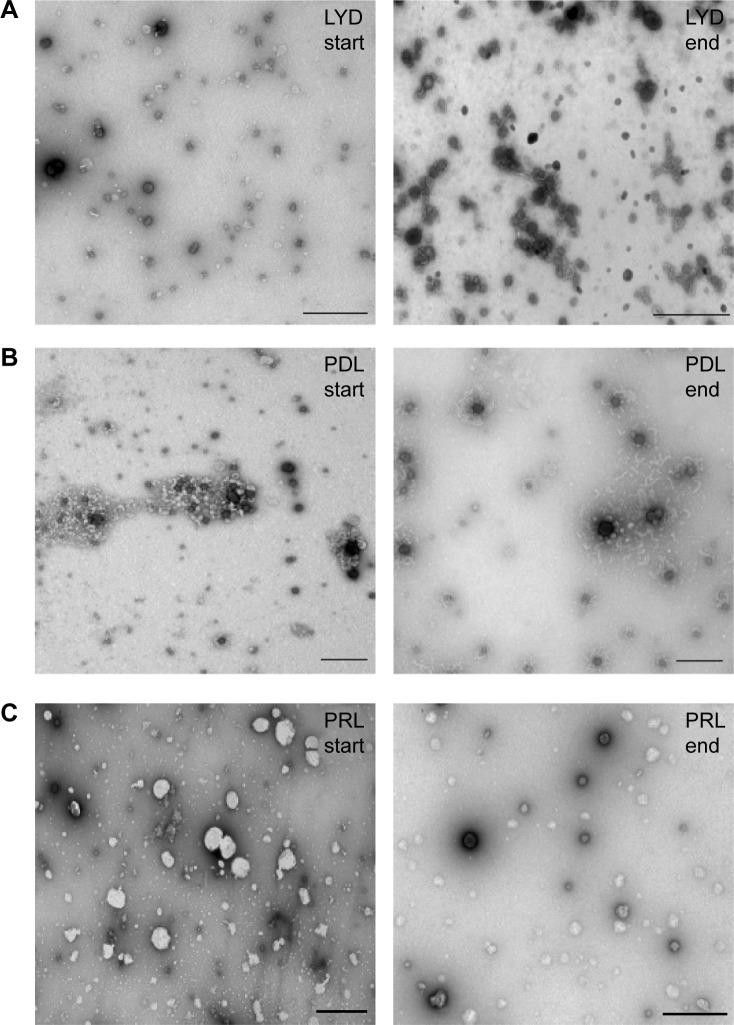
Electron microscopy of nanocomplexes. **Notes:** Negative staining transmission electron microscopy was used to visualize (**A**) LYD nanoparticles before and after concentration, (**B**) PDL nanoparticles before and after concentration, and (**C**) PRL nanoparticles before and after concentration. Scale bar =500 nm for all nanoparticles. 300 g/L dextran was used to concentrate all three different nanoparticle formulations. **Abbreviations:** LYD, liposome 1,2-di-O-octadecenyl-3-trimethylammonium propane (DOTMA)/1,2-dioleoyl-sn-glycero-3-phosphoethanolamine (DOPE), peptide Y, and DNA; PDL, peptide Y, DNA, liposome L^AP^1; PRL, peptide Y or RVG-9R, siRNA, liposome L^AP^2.

The sizes and zeta potentials (Figure 1B) of LYD nanocomplexes were determined before and after concentrating with different dextran solutions (100–300 g/L). There was no statistical difference in either the size or the zeta potential before and after concentration (size ranges: 98–137 nm and zeta potential: +64–69 mV), indicating that this procedure does not alter these biophysical properties. All PDI measurements before and after concentration were <0.3, indicating monodisperse nanoparticle populations. Cationic (LYD) and anionic (PDL and PRL) nanoparticle formulations^16^,^21^ were further characterized by negative staining TEM to determine their shape and morphology before and after concentration using 300 g/L of dextran (Figure 2). Most nanocomplexes were spheres (Figure 2A–C), but with some rods (in LYD and PDL; Figure 2A and B) and some toroidal structures (Figure 2A and B). The majority of the spherical particles measured by TEM for each formulation were in the range determined by DLS with no obvious differences between formulations before and after concentration.

This method of concentration has several advantages, as it preserves the biophysical characteristics of the nanocomplexes, avoids aggregation of the nanoparticles, and is a quick and simple method that does not need any specialized equipment.^28^ Based on the efficacy of the concentration, the short but predictable time required, and the fact that there were no major biophysical changes to the nanocomplexes before and after, we decided to use a dextran concentration of 300 g/L in ongoing experiments (unless otherwise stated).

### Concentration of LPD nanoparticles maintains their functional abilities

**Figure 3:**
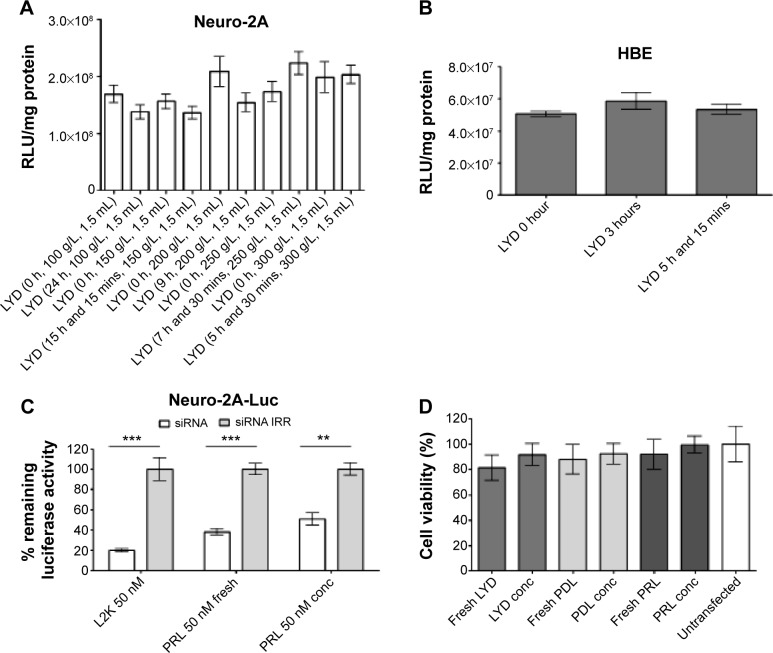
In vitro transfections with concentrated nanocomplexes retain transfection efficiency with lack of cytotoxicity. **Notes:** (**A**) A total of 1.5 mL of LYD nanocomplexes were concentrated using different amounts of dextran (100–300 g/L) and were used in luciferase transfections in Neuro-2A cells. (**B**) LYD nanoparticles before and after concentration (concentrated for 3 hours and 5 hours, 15 minutes) were used in luciferase transfections in HBE cells. (**C**) siRNA silencing from anionic PRL nanocomplexes (with peptide Y) before or after concentration made at a 4:3:1 molar charge ratio using siRNA targeting luciferase in Neuro-2A-Luc cells at 50 nM. 24 hours later, luciferase assays were performed. L2K/siRNA nanocomplexes were used as a positive control in all in vitro silencing experiments. (**D**) Viability of Neuro-2A cells following transfection for 24 hours with cationic LYD and anionic PDL and PRL nanocomplexes. Cationic nanocomplexes were made at a weight ratio of 1:4:1 (liposome:peptide:DNA) and the anionic nanocomplexes at a molar charge ratio of 4:3:1 (liposome:peptide:siRNA). The viability values were normalized to the untransfected control cells. The dextran concentration in the counter-dialyzing solution was kept constant (300 g/L) in Figure 3B–D. All transfections were performed in groups of six and mean values were calculated. Asterisks indicate comparisons of specific formulations with statistical significance (**P<0.01; ***P<0.001). **Abbreviations:** RLU, relative light units; LYD, liposome 1,2-di-O-octadecenyl-3-trimethylammonium propane (DOTMA)/1,2-dioleoyl-sn-glycero-3-phosphoethanolamine (DOPE), peptide Y, and DNA; HBE, human bronchial epithelial cells 16HBE14o–; siRNA, small interfering RNA; siRNA IRR, irrelevant control small interfering RNA; L2K, Lipofectamine^®^ 2000; PRL, peptide Y or RVG-9R, siRNA, liposome L^AP^2; conc, concentrated; PDL, peptide Y, DNA, liposome L^AP^1; P, peptides; R, siRNA; D, DNA; h, hours.

LYD nanoparticles were concentrated using different amounts of dextran (100–300 g/L), and were then used for the transfection of Neuro-2A cells (Figure 3A). There was no statistical difference in the transfection efficiency between the formulations irrespective of the amount of dextran used, or if they were concentrated or nonconcentrated. The same was true when the LYD nanoparticles that were concentrated against 300 g/L of dextran were used for the transfection of HBE cells (Figure 3B).

The concentrated, anionic PRL nanoparticles (with peptide Y)^21^ were used for luciferase silencing of Neuro- 2A-Luc cells (Figure 3C). All anionic formulations resulted in significant silencing between 45%–70%, and there was no difference between concentrated and nonconcentrated samples. The cell viability showed no particular difference between all the formulations tested, which included cationic LYD and anionic PDL and PRL made fresh or following concentration (Figure 3D).

**Figure 4:**
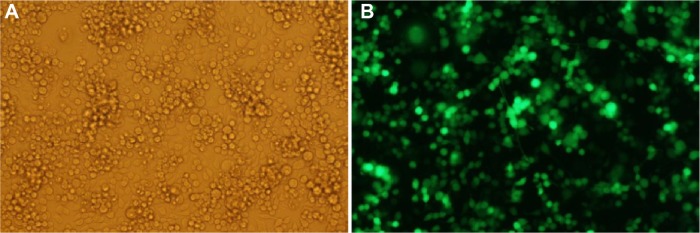
GFP transfection efficiency of nanocomplexes following concentration. **Notes:** One anionic PRL formulation was concentrated (300 g/L dextran) and then transfected Neuro-2A cells in serum-containing media. GFP expression was observed by epifluorescence microscopy 48 hours later. Representative cells are shown in (**A**) phase contrast and (**B**) transfected cells appear green (10× magnification). **Abbreviations:** GFP, green fluorescent protein; PRL, peptide Y or RVG-9R, siRNA, liposome L^AP^2.

The transfection efficiency of an anionic polyethylene glycol (PEG)ylated PDL formulation was evaluated with the plasmid expressing enhanced GFP in Neuro-2A cells. Fluorescent microscopy images provided evidence of the high transfection efficiency of PDL anionic nanoparticles following concentration (Figure 4).

### Ex vivo and in vivo administration of RVG-containing nanocomplexes

We then evaluated an LPR formulation for brain delivery using RVG-9R, which binds the nicotinic acetylcholine receptor,^30^ formulated with anionic liposomes and siRNA against the neuronal enzyme beta-secretase (BACE1). This enzyme cleaves the amyloid precursor protein to generate amyloid-beta peptides, which are landmarks in Alzheimer’s disease pathophysiology.^31^,^32^ BACE1 is therefore a main therapeutic target for Alzheimer’s disease.

**Figure 5:**
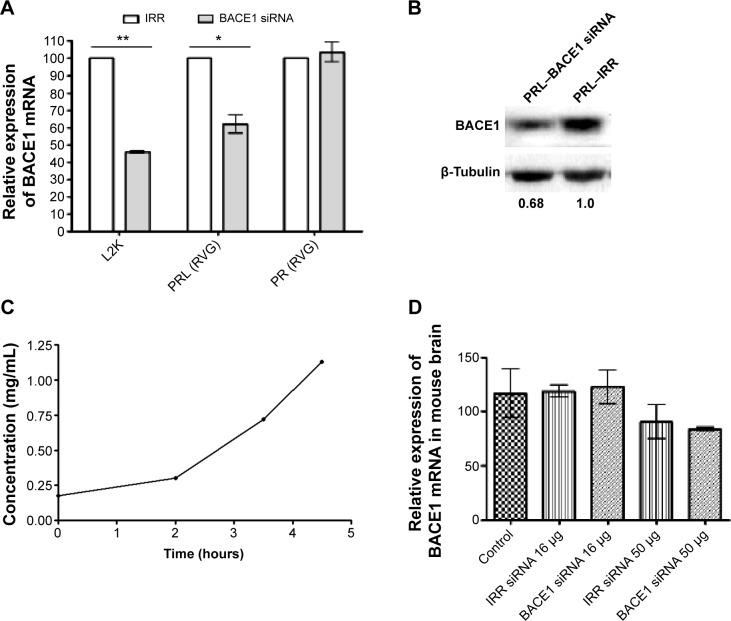
Nanocomplexes achieve silencing ex vivo, but not in the brain following intravenous administration. **Notes:** Mice brain explants were transfected ex vivo with peptide/siRNA complexes (PR: RVG-9R/BACE1siRNA; 4:1 weight ratio), anionic PEGylated PRL nanocomplexes with BACE1 siRNA, L2K, or complexes with irrelevant control siRNA (all at 100 nM), and then 48 hours post-transfection, the tissues were processed for analysis (**A**) by qRT-PCR and (**B**) by Western blot analysis of the BACE1 protein. Protein silencing was calculated with densitometric analysis using tubulin as loading control. (**C**) Anionic PRL nanocomplexes containing RVG-9R and BACE1 siRNA were concentrated using 300 g/L dextran over 3.5 hours, and this concentrated nanoparticle formulation was used (**D**) in intravenous injections. Mice were injected with 100 μL of anionic PRL nanoparticles containing 16 μg or 50 μg BACE1 siRNA or IRR siRNA, and 48 hours later, brains were processed for qRT-PCR analysis. The values are the means of three animals ± standard deviation. Asterisks indicate comparisons of specific formulations with statistical significance (*P<0.05; **P<0.01). **Abbreviations:** mRNA, messenger RNA; IRR, irrelevant control; siRNA, small interfering RNA; L2K, Lipofectamine^®^ 2000; PRL, peptide Y or RVG-9R, siRNA, liposome L^AP^2; RVG, rabies virus glycoprotein targeting peptide; PR, RVG-9R/BACE1siRNA; PEG, polyethylene glycol; qRT-PCR, quantitative reverse transcription polymerase chain reaction.

Ex vivo silencing studies were first performed with brain explant tissue transfected with 100 nM siRNA in anionic PRL nanoparticles containing RVG-9R or PR nanoparticles containing RVG-9R and siRNA (RVG-9R/siRNA). The analysis of silencing by qRT-PCR revealed about 40% silencing of *BACE1* with PRL nanoparticles (*P*<0.05) and no silencing was achieved with RVG-9R/BACE1R complexes (Figure 5A). L2K, which was used as a positive control, achieved 54% silencing compared to its irrelevant siRNA control (*P*<0.01). Western blotting revealed 32% silencing of BACE1 protein (Figure 5B). We then progressed to in vivo studies with just RVG-9R-targeted anionic PRL nanoparticles.

PRL nanocomplexes were then prepared for in vivo use by formulating initially at a concentration of 0.18 mg/mL BACE1 or irrelevant siRNA and then concentrated by dialysis against 300 g/L dextran over 4.5 hours, achieving an over sixfold concentration (Figure 5C). These nanoparticles were used for all the subsequent in vivo studies in mice and rats. The size and charge of concentrated BACE1 siRNA formulations were 79.9±2.95 nm and −44.8±1.5 mV, respectively, while the values for irrelevant control siRNA PRLs were similar at 73.3±4.0 nm and −36.9±2.3 mV.

Concentrated nanoparticles were administered intravenously into mice, and 48 hours later, the brains were assessed for silencing. No silencing of mouse *BACE1* was detected following intravenous injections (Figure 5D), unlike other reports that showed silencing even when the RVG/siRNA were delivered by intravenous injections.^30^,^33^–^35^ Other recent research showed nanoparticle deposition in the brain, but it did not examine the silencing effects there,^36^ or it assessed increased enzymatic activity following β-galactosidase administration.^37^ The lack of silencing following the intravenous administration of RVG-containing nanoparticles could be due to differences in both the amount of dose and the number of doses delivered, as we used a single 16 μg or 50 μg dose, whereas others use 50–150 μg for up to four doses.^30^,^33^–^35^ In addition, the nanoparticles used here are formed by self-assembly, whereas others use conjugation of the peptide by covalent bonds on the surface of the delivery vectors.^36^,^37^ However, this does not imply that our RVG peptide-containing nanoparticles did not pass the blood–brain barrier, as we did not examine their biodistribution.

**Figure 6:**
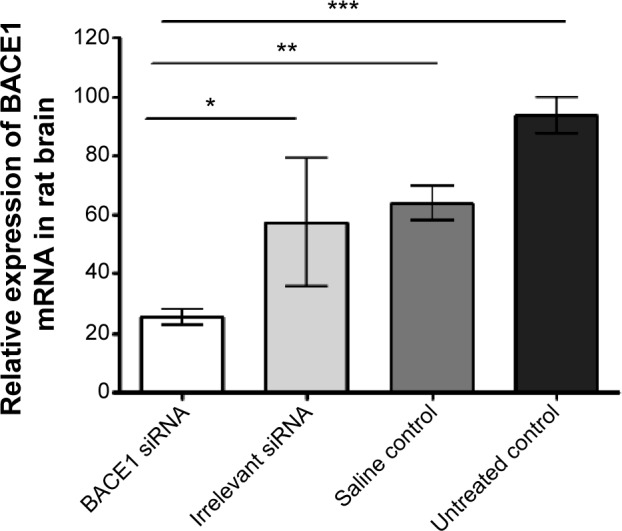
In vivo silencing of BACE1 following CED administration of PEGylated concentrated nanoparticles or control saline into rat striatum. **Notes:** Concentrated anionic PEGylated PRL nanoparticles containing RVG-9R peptide and BACE1 siRNA or irrelevant control siRNA were administered by CED in the striatum of rats, and 48 hours postadministration, tissues were removed for qRT-PCR analysis of siRNA-induced silencing of the *BACE1* gene. Values are the means of five animals ± standard deviation (n=3 for the untreated control animals and n=4 for the saline control animals) with one-way analysis of variance and Bonferroni’s post hoc analysis performed to calculate significant differences (*P<0.05; **P<0.01; ***P<0.001). **Abbreviations:** mRNA, messenger RNA; siRNA, small interfering RNA; CED, convection-enhanced delivery; PEG, polyethylene glycol; PRL, peptide Y or RVG-9R, siRNA, liposome L^AP^2; qRT-PCR, quantitative reverse transcription polymerase chain reaction; n, number.

We then evaluated direct brain administration in rats of anionic PRL nanoparticles (with RVG-9R) incorporating either BACE1 siRNA or irrelevant control, administered by CED^38^,^39^ into rat striata, and *BACE1* expression was examined 48 hours later. A significant reduction in *BACE1* mRNA was observed (Figure 6) between the *BACE1*-treated group and the control groups (*P*<0.05 compared to the irrelevant control group; *P*<0.01 compared to the saline group; and *P*<0.001 compared to the untreated control group).

Another study also performed direct brain administrations rather than using the intravenous route with RVG/siRNA complexes, and these showed good brain biodistribution and silencing of their target gene.^40^ The direct method avoids the problems of circulation clearance by the first-pass organs and the reticuloendothelial system, and requires less siRNA compared to the reported 50 μg^30^,^34^,^35^ or 150 μg doses.^33^ We have previously shown that anionic nanoparticles achieved more widespread dispersal in the brains of rats than their cationic counterparts when delivered by CED directly into the corpus callosum or striatum^15^,^16^ due to their lower affinity for anionic cell surface glycoproteins, and have therefore explored the potential of anionic siRNA nanocomplexes for neurodegenerative diseases. However, with CED, only small volumes (~5 μL in rat and ~120 μL in pig brains)^41^ can be delivered in vivo, therefore concentrating the nucleic acid-containing nanoparticles is necessary in order to deliver higher doses. Further studies using multiple siRNA dosing in order to investigate the duration of the silencing effect may achieve high levels of silencing for a sustained period using systemic or direct administration.

## Conclusion

In this study, we described the development of a method to concentrate cationic and anionic DNA and siRNA nanoparticles to enhance their utility for in vivo applications. Nanoparticles retained their biophysical properties and demonstrated similar silencing efficiency and DNA transfection efficiency in vitro to freshly prepared, nonconcentrated nanoparticles, all without cytotoxicity. Direct delivery into rat brains of *BACE1* siRNA demonstrated significant gene silencing. This method of concentrating nanoparticles for transfection could be used for other formulations, potentially enhancing their utility for in vivo applications.

## Figures and Tables

**Table 1:** Starting nanoparticle volumes, dextran concentrations used, total time of concentration, and concentration factors achieved for LYD formulations

Volume (mL)	Dextran concentration (g/L)	Time (hours)	Fold concentration
1.5	100	24	2.2
1.0	100	24	2.1
0.5	100	9	2.6
1.5	200	9	2.7
1.0	200	3.75	2.6
0.5	200	3.25	3.2
1.5	300	5.15	23.9
1.0	300	4	20.2
0.5	300	2.2	8.9

**Abbreviation:** LYD, liposome 1,2-di-O-octadecenyl-3-trimethylammonium propane (DOTMA)/1,2-dioleoyl-sn-glycero-3-phosphoethanolamine (DOPE), peptide Y, and DNA.
